# FMO1 Is Involved in Excess Light Stress-Induced Signal Transduction and Cell Death Signaling

**DOI:** 10.3390/cells9102163

**Published:** 2020-09-24

**Authors:** Weronika Czarnocka, Yosef Fichman, Maciej Bernacki, Elżbieta Różańska, Izabela Sańko-Sawczenko, Ron Mittler, Stanisław Karpiński

**Affiliations:** 1 Department of Botany, Institute of Biology, Warsaw University of Life Sciences, Nowoursynowska 159, 02-776 Warsaw, Poland; elzbieta_rozanska@sggw.edu.pl (E.R.); izabela_sanko_sawczenko@sggw.edu.pl (I.S.-S.); 2 Department of Plant Genetics, Breeding and Biotechnology, Institute of Biology, Warsaw University of Life Sciences, Nowoursynowska 159, 02-776 Warsaw, Poland; maciej_bernacki@sggw.edu.pl (M.B.); stanislaw_karpinski@sggw.edu.pl (S.K.); 3 The Division of Plant Sciences and Interdisciplinary Plant Group, College of Agriculture, Food and Natural Resources, Christopher S. Bond Life Sciences Center University of Missouri, Columbia, MO 65211, USA; fichmany@missouri.edu (Y.F.); mittlerr@missouri.edu (R.M.); 4 Institute of Technology and Life Sciences, Falenty, Al. Hrabska 3, 05-090 Raszyn, Poland; 5 Department of Surgery, University of Missouri School of Medicine, Columbia, MO 65212, USA

**Keywords:** flavin-dependent monooxygenase 1, SAA, SAR, cell death, Arabidopsis

## Abstract

Because of their sessile nature, plants evolved integrated defense and acclimation mechanisms to simultaneously cope with adverse biotic and abiotic conditions. Among these are systemic acquired resistance (SAR) and systemic acquired acclimation (SAA). Growing evidence suggests that SAR and SAA activate similar cellular mechanisms and employ common signaling pathways for the induction of acclimatory and defense responses. It is therefore possible to consider these processes together, rather than separately, as a common systemic acquired acclimation and resistance (SAAR) mechanism. *Arabidopsis thaliana* flavin-dependent monooxygenase 1 (FMO1) was previously described as a regulator of plant resistance in response to pathogens as an important component of SAR. In the current study, we investigated its role in SAA, induced by a partial exposure of *Arabidopsis* rosette to local excess light stress. We demonstrate here that *FMO1* expression is induced in leaves directly exposed to excess light stress as well as in systemic leaves remaining in low light. We also show that FMO1 is required for the systemic induction of *ASCORBATE PEROXIDASE 2* (*APX2*) and *ZINC-FINGER OF ARABIDOPSIS 10* (*ZAT10*) expression and spread of the reactive oxygen species (ROS) systemic signal in response to a local application of excess light treatment. Additionally, our results demonstrate that FMO1 is involved in the regulation of excess light-triggered systemic cell death, which is under control of LESION SIMULATING DISEASE 1 (LSD1). Our study indicates therefore that FMO1 plays an important role in triggering SAA response, supporting the hypothesis that SAA and SAR are tightly connected and use the same signaling pathways.

## 1. Introduction

Because of their sessile nature, in the course of evolution plants have evolved systemic defense mechanisms in response to many different stresses, including pathogens, wounding and abiotic stresses, which are essential for plant survival in unfavorable conditions. One of these mechanisms is systemic acquired resistance (SAR), in which, upon local pathogen attack, plants induce protection against a subsequent infection, leading to broad-spectrum disease resistance at distal, uninfected tissues. SAR mechanism involves transcriptional reprogramming of various defense-related genes (such as pathogenesis-related (PR) genes) [1], rapid production of reactive oxygen species (ROS) and nitric oxide [2], accumulation of the salicylic acid (SA) [3], and subsequent hypersensitive death of infected cells in order to restrain pathogens spread [4].

However, not only pathogens can evoke systemic signal transduction. For instance, excess- or high-light treatment of one leaf is able to induce the systemic signal that is rapidly transmitted to distal leaves, resulting in an acclimatory response and enhanced tolerance to the following stress, called systemic acquired acclimation (SAA) [5]. Recent studies identified several different signaling processes responsible for SAA, such as transcriptomic reprograming [6], systemic changes in ROS and phytohormone levels [7,8], changes in Ca^2+^ [9], in electrical signaling [10] and in non-photochemical quenching (NPQ) [11,12]. It was also shown that spatial-temporal SAA induction is dependent on direct vascular connections [13]. There is growing evidence that the SAA signal is transduced by bundle sheath parenchyma cells [14]. For instance, high-light treatment is able to induce specific changes in the bundle sheath parenchyma cells plasma membrane electrical potential, called photoelectrophysiological signaling (PEPS) and physical interruption of bundle sheath cells layer blocked PEPS and SAA induction [10]. PEPS was shown to be involved not only in SAA, but also in the immune defense in response to pathogens [10]. In *Arabidopsis thaliana,* robust molecular markers of SAA, ASCORBATE PEROXIDASE 2 (APX2, AT3G09640) and ZINC-FINGER OF ARABIDOPSIS 10 (ZAT10, AT1G27730) [5,15], are expressed in bundle sheath parenchyma cells [16,17]. Moreover, the speed of the PEPS between different leaves depends on *APX2* gene expression [10]. Both APX2 and ZAT10 respond to ROS and are key regulators of plant stress signaling pathways [16,18,19]. Moreover, the transcription factor ZAT10 plays a key role in the specific activation of the ROS-related antioxidant system, including APX2 [20].

Although SAR and SAA were previously considered as separate processes, more and more evidence indicates several common elements between them. For instance, the systemic ROS wave, SA, ethylene (ET), and brassinosteroid signaling, as well as PEPS, are suggested as joint mechanisms of SAA and SAR [21]. Light stress “priming” treatment was shown to confer long-term stress acclimation not just to a second high-light exposure, but is also able to induce cross-tolerance against bacteria [10,22]. Initiation of SAR has been shown to occur in a light-dependent manner and the SAR mechanism differ under variable light regimes [23]. Both SAR and SAA induction is normally associated with programmed cell death (PCD) induction in some cells. PCD is a highly regulated and organized process of cell suicide, which plays an important role in the cell homeostasis maintenance, tissue specialization, removal of damaged or infected cells and the acclimation response [24]. Cell death in plants is preceded by the production and accumulation of ROS, which causes redox homeostasis disturbance and damage to cellular components, such as membrane lipids, nucleic acids and proteins. However, during SAR and SAA ROS play also an important role as signaling molecules [8]. Moreover, cell death was proven to be regulated by LESION SIMULATING DISEASE 1 (LSD1), ENHANCED DISEASE SUSCEPTIBILITY 1 (EDS1) and PHYTOALEXIN DEFICIENT 4 (PAD4) [25,26]. These proteins were originally defined as immune defense response regulators [25]. Later on, it was demonstrated that they also regulate the acclimation to high-light, UV, photorespiration and lysigenous aerenchyma formation [26,27,28].

FLAVIN-DEPENDENT MONOOXYGENASE 1 (FMO1, AT1G19250) belongs to the family of flavin containing monooxygenases (FMO), consisting of 29 members in *Arabidopsis* [29,30]. FMOs are present in all kingdoms of life, all possessing a flavin–adenine dinucleotide (FAD) prosthetic group and use NAD(P)H and O_2_ to oxidize low-molecular-weight molecules [31]. FMO1 is a positive defense regulator and was firstly described to be involved in response to bacterial and oomycete pathogens in *Arabidopsis* and powdery mildew fungus in barley [29,32,33,34]. More recent works showed that *Arabidopsis* FMO1 is also needed in the response to oviposition by the Large White butterfly [35] and belowground plant-to-plant signaling mechanism in response to oviposition to prepare for potential pathogen invasion [36]. The *Arabidopsis* knockout line of FMO1 (*fmo1*) is impaired in the establishment of SAR, unable to initiate systemic accumulation of SA and systemic expression of defense genes [34]. The transcription reprogramming of systemic leaves during SAR is also depends on a functional FMO1 [1,37]. Upon bacterial infection, *fmo1* mutant fails to form lesions and allows pathogen invasive growth, but introducing wild type *FMO1* complements the *fmo1* defects in pathogen resistance [32]. However, the responses at the site of pathogen attack, including increases in the SA, jasmonic acid and camalexin levels, and expression of defense related genes, are activated in *fmo1* in a similar manner to that of wild type [34]. Induction of FMO1 expression at the site of pathogen infection is attenuated by the recessive mutations in *EDS1* and *PAD4* genes [34]. The FMO1-dependent promotion of SAR relies on EDS1/PAD4 activities and FMO1 positively regulates the EDS1-driven cell death pathway [32]. While the *fmo1* mutant shows enhanced susceptibility to pathogens and loss of SAR [32,34], *FMO1* overexpression increases the resistance against bacterial and oomycete pathogens [33,38]. There is also evidence that expression of *FMO1* is induced by many stress factors, such as hypoxia [39] or upon superoxide generation [29]. Therefore, FMO1 was proposed as a marker gene for defense and cell death in plants [29]. Furthermore, it was shown that *FMO1* expression is reduced in non-stressed plants deficient in the positive regulators of cell death, EDS1 and PAD4 [40,41]. Meanwhile, plants with a mutation in the negative regulator of cell death, LSD1, demonstrated elevated *FMO1* expression [29,40,42], which suggests that FMO1 is involved in the LSD1-, EDS1- and PAD4-dependent cell death regulation.

In the work by Mishina and Zeier (2006) it was suggested that FMO1 is involved in the synthesis of a yet unknown metabolite required for the transduction or amplification of a signal during the early phases of systemic signal transduction [34]. Such biochemical function of FMO1 was recently discovered independently by two research groups. FMO1 was shown to function as a pipecolic acid (Pip) *N*-hydroxylase, catalyzing the biochemical conversion of Pip to a previously undescribed plant metabolite, *N*-hydroxy-pipecolic acid (*N*-OH-Pip, NHP) in the presence of FAD and NADH as cofactors [43,44]. NHP is a mobile molecule initiating SAR signal transduction and exogenously applied NHP moves systemically in *Arabidopsis* and rescues the SAR-deficiency of *fmo1* mutant [43,44]. However, the signaling pathway or molecule responsible for the activation of *FMO1* expression is yet to be specified.

Because the mechanisms of SAR and SAA employ several common molecular elements and pathways, it is possible that FMO1, considered as exclusively SAR regulator, is also an important regulator of SAA. Moreover, since the expression of *FMO1* is highly induced in the *lsd1* mutant [40], it is further possible that LSD1 and FMO1 regulate at least some common pathways, such as ROS accumulation and cell death. Our results demonstrate that indeed FMO1 is involved in SAA and is a positive regulator of cell death propagation in *lsd1* mutant. Thus, we report that LSD1 and FMO1 are interconnected, as well as provide evidence that FMO1 is yet another common element of SAR and SAA, collectively called SAAR [14].

## 2. Materials and Methods

### 2.1. Plant Material and Growth Conditions

*Arabidopsis thaliana fmo1-1* (SALK_026163) [32], *lsd1-1, lsd1-1/fmo1-1* mutants and a line overexpressing *FMO1* gene, all in Columbia-0 (Col-0) background were used in this study along with Col-0 wild type plants. The expression of *FMO1* in *fmo1*, *lsd1*, *lsd1/fmo1* and *FMO1-OE* was checked with real-time quantitative PCR (qPCR) prior experiments (Supplementary Figure S1). Seeds were sown on Jiffy pots, stratified for two days in 4 °C and germinated in standard laboratory conditions (8 h/16 h day/night, photosynthetic photon flux density 100 ± 25 µmol photons m^−^^2^ s^−^¹, 50% relative air humidity and temperature 22/18 °C day/night).

### 2.2. Plasmid Construction

In order to obtain genetic construct for *FMO1* overexpression, total plant RNA was extracted from three-week-old wild type plants using the TRIzol reagent (Invitrogen, Life Technologies, Carlsbad, CA, USA) and purified from residual DNA with a DNA-free™ DNA Removal Kit (Ambion, Life Technologies, Carlsbad, CA, USA). The total RNA concentration was measured at 260 nm using a UV–VIS spectrophotometer (NanoDrop, Thermo Fisher Scientific, Waltham, MA, USA). cDNA synthesis was performed on 2 μg of RNA using a High Capacity cDNA Reverse Transcription Kit (Life Technologies, Carlsbad, CA, USA). Full-length *FMO1* (AT1G19250) coding sequence was amplified by a polymerase chain reaction (PCR) using a Phusion HighFidelity DNA Polymerase (Thermo Fisher Scientific) on cDNA template with specific primers, extended with the attB sites for Gateway cloning (Invitrogen). Primer sequences are provided in Supplementary Table S1. The genetic construct, in which GFP-FMO1 fusion was expressed under the control of the cauliflower mosaic virus 35S promoter (p35S: GFP-FMO1) was obtained by recombinational Gateway cloning (Invitrogen) using pK7WGF2 vector [45].

### 2.3. Stable Transformation of Arabidopsis

In order to generate *Arabidopsis* plants overexpressing FMO1, the construct was transformed into plants using *Agrobacterium tumefaciens* strain C58C1 by floral dip protocol [46]. Selection for kanamycin-resistance and homozygous plant production were performed as previously described [47].

### 2.4. Excess Light (EL) Treatment

Four-week-old rosettes were taken for analysis. For qPCR analysis, the EL (1700 µmol photons m^−2^ s^−1^) was applied for 2 min on 6th leaf (designated as local leaf, LL) without generation of radiant heat, using a KL 2500 LED white light source and a fiber optic cable (SCHOTT AG, Mainz, Germany). Local and systemic leaves (3rd and 9th), having vascular connection [13] were harvested before stress, immediately after EL treatment as well as after 10 and 20 min of recovery. For cell death analysis local leaves were exposed to 10-minute-long EL stress, and local and systemic leaves were collected before and 1 day after stress (for micro-lesion analysis) and, additionally, 3 and 6 days after EL treatment (for ion leakage analysis).

### 2.5. RNA Extraction and cDNA Synthesis

RNA was extracted from local and systemic leaves separately in five biological replicates. Each biological replicate consisted of leaves harvested from three individual rosettes. Total RNA extraction was performed using GeneMATRIX Universal RNA Purification Kit (EURX, Gdańsk, Poland) with additional step of on-column DNaseI digestion. RNA concentration and purity were analyzed spectrophotometrically with Eppendorf BioSpectrometer (Eppendorf, Hamburg, Germany). The RNA quality was tested by electrophoretic separation in 1% agarose gel. cDNA synthesis was performed for equimolar RNA amounts of each sample using a High Capacity cDNA Reverse Transcription Kit (Thermo Fisher Scientific).

### 2.6. Relative Gene Expression Measurement by Real-Time qPCR

Real-time qPCR was performed for cDNA obtained from non-treated plants, excess-light treated local leaves and systemic leaves. qPCR was performed in duplicates for each of five biological replicates, using the CFX Connect Real-Time PCR Detection System (Bio-Rad Laboratories, Hercules, CA, USA) and Power SYBR Green Master Mix (Thermo Fisher Scientific). The following cycling program was used in qPCR: 95 °C for 10 min, followed by 40 cycles of denaturation in 95 °C for 15 s and annealing/extension in 60 °C for 30 s. Primers were designed with Primer3Plus software (Primer3Plus, Free Software Foundation, Inc., Boston, MA, USA) and sequences are provided in Supplementary Table S1. Two reference genes were used, according to the RefGenes tool incorporated in Genevestigator [48], *5-FORMYLTETRAHYDROFOLATE CYCLOLIGASE* (*5-FCL*, AT5G13050) and *PROTEIN PHOSPHATASE 2A SUBUNIT A2* (*PP2AA2*, AT3G25800). The specificity of each primer pair was analyzed by melting curve. The efficiency of real-time qPCR was calculated using LinRegPCR tool [49]. Calculation of relative gene expression levels and the significance of difference between tested samples was performed using CFX Maestro Software (Bio-Rad Laboratories).

### 2.7. ROS Imaging

Four-week-old plants were fumigated for 30 min in a glass container using a nebulizer (Punasi Direct, Hong Kong, China) with a solution of 50 µM H_2_DCFDA (Millipore-Sigma, St. Louis, MO, USA) in 0.05 M phosphate buffer, pH 7.4, 0.01% (*v*/*v*) Silwet L-77 [50,51]. Following the fumigation, a local EL stress treatment (1700 µmol photons m^−2^ s^−1^) was applied to a single local leaf for 2 min. Oxidized DCF fluorescence was detected at excitation/emission 490 nm/520 nm using IVIS Lumina S5 (PerkinElmer, Waltham, MA, USA). Images were acquired every 2 min up to 30 min after the stress. ROS accumulation was analyzed using Living Image 4.7.2 software (PerkinElmer). Time-course images were generated and radiant efficiency of regions of interest (ROI) was calculated. Each data set includes standard error for 32 biological repeats and was assigned a Student *t*-test score.

### 2.8. Cell Death Quantification by Electrolyte Leakage Analysis

Cell death was quantified by ion leakage from whole rosettes for 6 plants *per* genotype in two independent experiments (*n* = 12). The 6th leaf of four-week-old plants was treated with EL for 10 min. Plants 3 days post treatment (3 dpt) and 6 days post treatment (6 dpt) together with non-treated counterparts were harvested and transferred to 50 mL Falcon tubes filled with 35 mL of deionized water. The conductivity was measured with InoLab Cond Level 1 conductivity meter (WTW Wissenschaftlich-Technische Werkstätten GmbH, Weilheim, Germany) and calculated as previously described [26,52].

### 2.9. Trypan Blue Staining and Micro-Lesions Counting

Trypan blue (TB) stock (30 µmol trypan blue; Sigma Aldrich St. Louis, Mo, USA) in a mixture of lactic acid, glycerol and water (10 mL: 10 mL: 20 mL) was diluted with 96% ethanol (1:2) to obtain TB working solution. Leaves were harvested from non-treated plants and plants 24 h after EL treatment. Twelve non-treated, local and systemic leaves *per* each genotype, from two independent experiments (*n* = 24), were harvested and dipped separately in TB working solution in 50 mL Falcon tubes. Leaves were incubated in TB working solution for 30 min in room temperature and gently shaken from time to time. After incubation, the TB working solution was removed and replaced witch methanol. The leaves were incubated in methanol for 24 h. During the incubation, methanol was changed several times for the fresh one. Leaves devoid of chlorophyll were visualized using Nikon SMZ18 stereomicroscopes (Nikon Inc., Melville, NY, USA) with an adapted camera Nikon d5100 (Nikon Inc.). Photos of individual leaves were analyzed in ImageJ software version 1.8.0 (http://rsb.info.nih.gov/ij) and blue spots (micro-lesions) were counted per mm^2^ of leaf area.

## 3. Results

### 3.1. FMO1 Expression Is Induced in Both Local EL-Treated and Systemic Leaves

**Figure 1:**
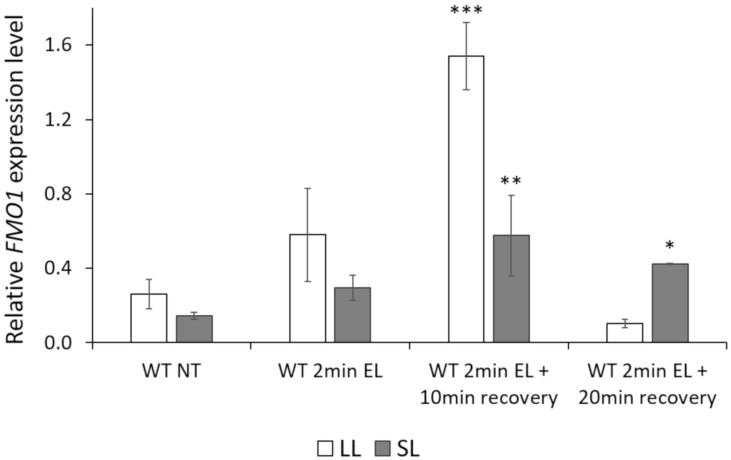
Relative expression levels of *FMO1* in wild type (WT) *Arabidopsis thaliana* in non-treated (NT) plants, after 2 min of EL treatment, and after 10 and 20 min of recovery. Values ± SD are averages for 5 independent biological replicates, for which qPCRs were performed in duplicate (*n* = 10). Stars above the bars indicate statistically significant difference, in comparison to the wild type, according to the t-test at a level of *p* < 0.05 (*), *p* < 0.005 (**) or *p* < 0.001 (***).

To test whether the *FMO1* gene is induced upon EL treatment in local (LL) and/or untreated (systemic, SL) leaves we performed a time-course qPCR analysis for non-treated, as well as EL-treated, local leaves and systemic leaves remaining in low light. Our results indicated that *FMO1* expression was induced, although not statistically significantly, both locally and systemically already after the 2 min long EL treatment (Figure 1). The highest induction of *FMO1* expression was observed 10 min after recovery in both local and systemic leaves, whereas, after 20 min of recovery, the *FMO1* expression level in both local and systemic leaves dropped. Nevertheless, after 20 min of recovery the systemic leaves remained statistically significant induction of *FMO1* expression, when compared to non-treated systemic leaves. These results indicate that *FMO1* expression is rapidly induced upon EL treatment in both local and systemic leaves.

### 3.2. FMO1, Together with LSD1, Is Required for the Spread of the ROS Systemic Signal

**Figure 2:**
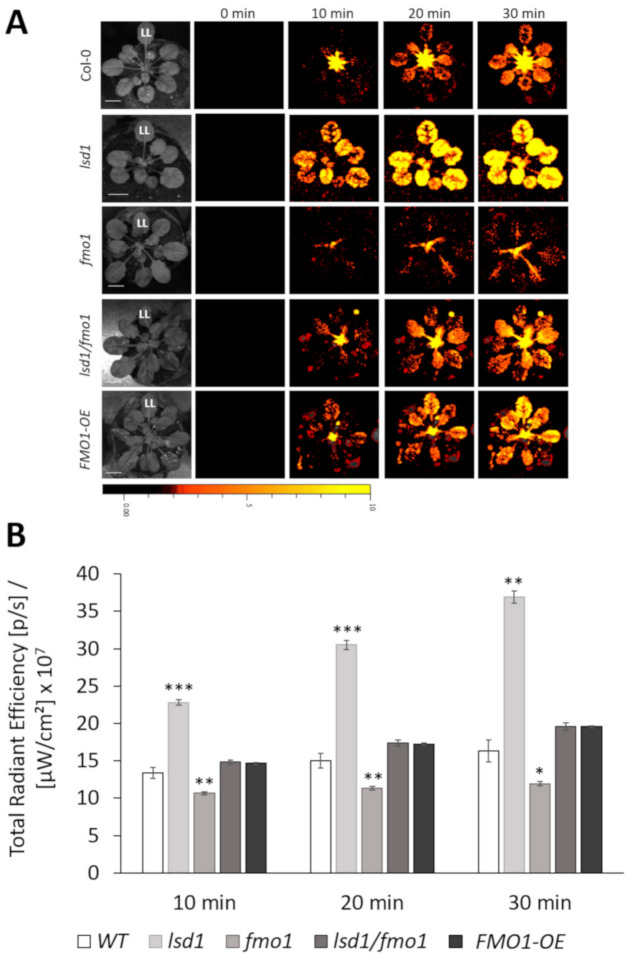
Time-lapse imaging of systemic ROS accumulation in wild type, *lsd1*, *fmo1, lsd1/fmo1* and *FMO1-OE Arabidopsis thaliana* plants subjected to a 2-minute-long local EL stress treatment, applied to local leaf (LL) only (**A**). Statistical analysis of ROS accumulation in local and systemic leaves at 10, 20 and 30 min (**B**). Values ± SD are averages of 8 rosettes for four independent experiments (*n* = 32). Stars above the bars indicate statistically significant difference, in comparison to the wild type, according to the t-test at a level of *p* < 0.05 (*), *p* < 0.005 (**) or *p* < 0.001 (***). Scale bar = 1 cm.

We next wanted to determine the role of FMO1 in spreading the EL stress signal to systemic leaves. Because ROS are among the fastest signal molecules spreading from leaf to leaf [8,53], we employed local EL treatment and subsequent whole-plant ROS accumulation imaging. ROS levels were monitored over the course of 30 min in the wild type, *fmo1* mutant and *FMO1* overexpressing plants (*FMO1-OE*). Since the LSD1 is known to control ROS accumulation upon stress [26], we also used *lsd1* mutant and *lsd1/fmo1* double mutant in order to analyze the FMO1 and LSD1 interdependence (Figure 2).

As shown in Figure 2, two-minute-long illumination of a single leaf resulted in a time-dependent ROS accumulation in both EL-treated and systemic leaves. As expected, *lsd1* mutant demonstrated faster ROS accumulation in both treated and untreated leaves, compared to the wild type. *lsd1* plants had significantly higher level of ROS, while *fmo1* mutant showed significantly lower ROS accumulation 10, 20 and 30 min after EL exposure. Interestingly, *lsd1/fmo1* double mutant proved to have similar levels of ROS, as the wild type plants, which means that *fmo1* mutation was able to revert *lsd1* hyper-ROS accumulating phenotype. These results indicate that FMO1 is a positive regulator of ROS propagation in the *lsd1* background, which means that FMO1 is required for the systemic spread of ROS that lies at the same signaling pathway as LSD1.

In order to assess which specific ROS are engaged in the systemic ROS spread, we performed a qPCR experiment in which we tested the relative expression levels of genes encoding specific ROS markers in non-stressed plants, EL-treated leaves (2 min EL + 10 min recovery) and corresponding systemic leaves. *Telomere-binding protein TRF-like 4* (*TRFL4*, AT3G53790), *beta-glucosidase BGLU23* (AT3G09260) and *DMR6-LIKE OXYGENASE 1* (*DLO1*, AT4G10500) were used as marker genes specifically responsive to singlet oxygen (^1^O_2_), superoxide anion (O_2_^−^) and hydrogen peroxide (H_2_O_2_), respectively [54]. Our results indicated that 2-minute-long EL exposure of local leaf was sufficient to significantly induce the expression of *TRFL4* in *lsd1* mutant (Supplementary Figure S2). Although not statistically significant, the expression levels of *TRFL4* were lower in *fmo1* mutant in both local and systemic leaves. After EL treatment, we observed an increase in *BGLU23* expression levels in all tested genotypes, which indicates that local EL exposure caused O_2_^−^ generation. In systemic leaves, the *BGLU23* expression levels were significantly elevated in *lsd1* mutant and reduced in *fmo1* plants, when compared to systemic leaves of the wild type. The transcription of H_2_O_2,_ marker gene, *DLO1*, proved to be elevated in all non-treated, local and systemic leaved of *lsd1* mutant. Meanwhile, *DLO1* expression was significantly reduced in EL-treated local and systemic leaves of *fmo1* and *lsd1/fmo1* double mutant, in comparison to wild type plants. Interestingly, systemic leaves of *FMO1-OE* line showed elevated *DLO1* transcript level, when compared to the wild type. These results indicate that the marker genes of all ^1^O_2_, O_2_^−^ and H_2_O_2_ were to some extent involved in the local response to EL. However, the most responding marker gene in tested genetic system was *DLO1*, indicating that H_2_O_2_ may be the main ROS form engaged in ROS signal propagation dependent on FMO1.

### 3.3. Functional FMO1 Is Required for the Systemic Induction of APX2 and ZAT10 Expression upon Local EL Treatment

APX2 and ZAT10 are well-described regulators of systemic acquired acclimation (SAA) [10,55]. The expression of *APX2* and *ZAT10* is induced upon excess-light treatment in both local and systemic leaves [5,15]. Therefore, we decided to test the expression of *APX2* and *ZAT10* in non-treated, EL-treated (2 min EL + 10 min recovery) and corresponding systemic leaves in the genotypes tested in the current study.

**Figure 3:**
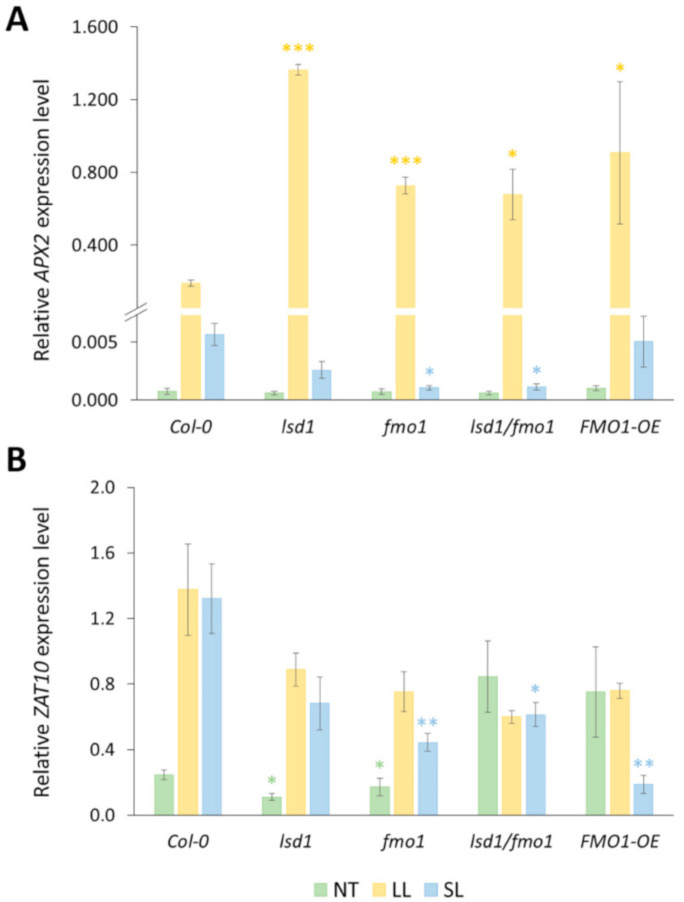
Relative expression of SAA marker genes, *APX2* (**A**) and *ZAT10* (**B**) in wild type, *lsd1*, *fmo1, lsd1/fmo1* and *FMO1-OE* *Arabidopsis thaliana* plants, performed for non-treated (NT) leaves, local leaves exposed to EL stress (LL) and systemic leaves (SL). Values ± SD are averages for 5 independent biological replicates, for which qPCRs were performed in duplicate (*n* = 10). Stars above the bars indicate statistically significant difference, in comparison to the wild type, according to the t-test at a level of *p* < 0.05 (*), *p* < 0.005 (**) or *p* < 0.001 (***).

There was no difference in *APX2* transcript levels among non-treated genotypes (Figure 3A). Upon EL treatment, *APX2* transcription was highly induced in local leaves of all tested genotypes. However, the levels of *APX2* transcript abundance were significantly higher in *lsd1*, *fmo1*, *lsd1/fmo1* mutants and *FMO1-OE* line, compared to the wild type plants. Interestingly, in the systemic leaves of *fmo1* and *lsd1/fmo1* mutants the increase in *APX2* expression was significantly lower, in relation to the wild type.

*ZAT10* expression levels in non-stress conditions were significantly decreased in *lsd1* and *fmo1* mutants (Figure 3B). After EL treatment *ZAT10* transcription was elevated in all the genotypes, except *lsd1/fmo1* double mutant and *FMO1-OE* line. In systemic leaves we observed a similar pattern to *APX2* expression, since *ZAT10* transcript abundance was lower in *fmo1* and *lsd1/fmo1* mutants, and additionally in *FMO1-OE* line. These results indicate that functional FMO1 is needed for systemic signaling inducing *APX2* and *ZAT10* expression.

### 3.4. FMO1 Is Involved in LSD1-Dependent Spread of Systemic Cell Death

LSD1 has been broadly described as a negative regulator of cell death spread, called runaway cell death (RCD) [8,24]. Upon stress, the *lsd1* mutant accumulates high levels of ROS (Figure 2 and [40]) and cannot restrain cell death propagation once it has been initiated [26,27,56]. Because we observed a reversion in the *lsd1* phenotype in terms of ROS accumulation, by introducing *fmo1* mutation into *lsd1* background (*lsd1/fmo1* double mutant), we set out to examine whether FMO1 is involved in a cell death pathway, negatively regulated by LSD1.

**Figure 4:**
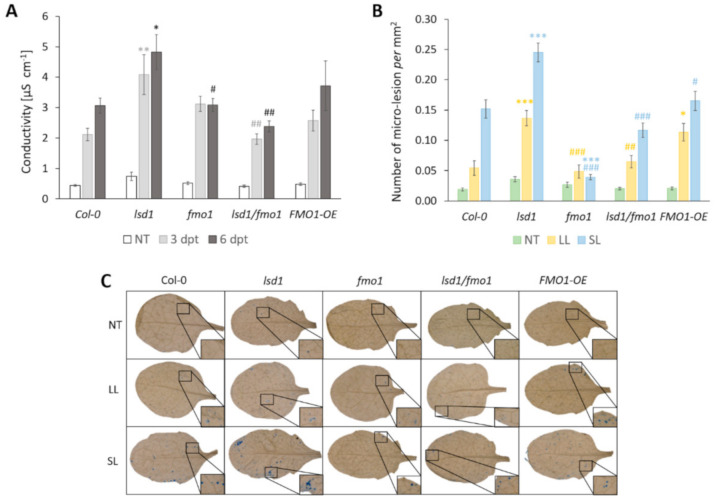
Cell death in wild type, *lsd1*, *fmo1, lsd1/fmo1* and *FMO1-OE* *Arabidopsis thaliana* plants, quantified as cellular electrolyte leakage from the whole rosettes in non-treated (NT) plants, plants 3 days post treatment (3 dpt) and 6 days post treatment (6 dpt). Values ± SD are averages of 6 rosettes for two independent experiments (*n* = 12) (**A**). Cell death quantified as micro-lesion number in non-treated (NT), local leaves exposed to EL stress (LL) and systemic leaves (SL). Values ± SD are averages of 12 leaves for two independent experiments (*n* = 24) (**B**). Symbols above the bars indicate the significant difference from the wild type (Col-0) at the level of *p* < 0.05 (*), *p* < 0.005 (**) or *p* < 0.001(***) or from *lsd1* mutant at the level of *p* < 0.05 (#), *p* < 0.005 (##) or *p* < 0.001(###) as indicated by the Tukey’s multiple comparison test. The representative pictures of micro-lesions in the wild type (Col-0), *lsd1*, *fmo1*, *lsd1/fmo1* mutants and *FMO1-OE* line in non-stress as well as local EL-treated leaves (LL) and systemic leaves (SL) (**C**).

We quantified the cell death in two ways. In the first method we used the measurement of total ion leakage from the rosettes [26,52,57] before stress, as well as 3 and 6 days after local EL treatment. Our results indicated that in non-stress conditions there were no significant changes in cell death among tested genotypes (Figure 4A). However, EL treatment of local leaf caused noticeable increase in ion leakage 3 days post treatment (dpt) in all tested lines. As expected, *lsd1* mutant showed significantly increased cell death, in comparison to wild type counterparts. However, most importantly, *lsd1/fmo1* double mutant demonstrated reduced ion leakage, when compared to the *lsd1* single mutant. The cell death spread 6 dpi was most visible in *lsd1* mutant (Figure 4A and Supplementary Figure S3). Nevertheless, we observed slower spread of cell death in *fmo1* and *lsd1/fmo1* mutants, when compared to the *lsd1* single mutant (Figure 4A and Supplementary Figure S3). These results indicate that the lack of functional FMO1 was able to revert the cell death spread in *lsd1* background.

The second method for cell death assessment we used was the staining of non-treated, EL-treated local leaves and systemic leaves with trypan blue in order to analyze the micro-lesion formation. Micro-lesions constitute small lesion area within the leaf tissue, comprising one or a few dead cells [58]. The development of micro-lesions in systemic leaves was previously shown only for SAR in response to pathogen attack [58,59]. So far, there was no experimental proof of the existence of micro-lesion formation during SAA. Our results demonstrate that a 10-minute-long local EL stress was generally able to induce micro-lesion formation 24 h after treatment in both local and systemic leaves (Figure 4B,C). Interestingly, we observed higher numbers of micro-lesions in systemic leaves than in local leaves. Nevertheless, *lsd1* mutant had significantly increased numbers of micro-lesions in both local and systemic leaves, while *FMO1-OE* line demonstrated higher micro-lesion number only in local leaves. However, most importantly, the formation of systemic micro-lesions in *fmo1* mutant was significantly inhibited, when compared to the wild type and again *lsd1/fmo1* double mutant manifested reversed micro-lesion formation phenotype in relation to the *lsd1* single mutant. These results suggest that FMO1 is a positive regulator of systemic cell death signal transmission and takes part in the LSD1-dependent cell death regulatory pathway.

### 3.5. Proposed Model of FMO1 Involvement in ROS Signaling, SAAR and Cell Death

**Figure 5:**
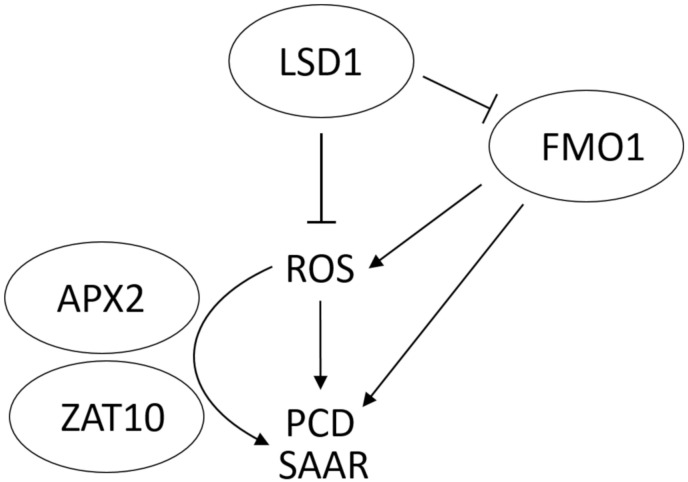
Proposed model of FMO1 involvement in ROS signaling, systemic acquired acclimation and resistance (SAAR), and cell death.

Based on the results presented in this work, we propose the following model of the FMO1 involvement in the ROS signaling, SAAR and cell death upon EL treatment (Figure 5). LSD1 negatively influences the FMO1-dependent signaling pathways. Among these pathways there is a propagation of ROS signals from local, exposed to EL leaves, to systemic leaves, as well as APX2- and ZAT10-dependent signaling. ROS spread, positively regulated by FMO1, leads to cell death and simultaneously to SAAR.

## 4. Discussion

The role of FMO1 has been well described thus far in the SAR pathway [32,33,34]. Taking into consideration many common signaling pathways between SAR and SAA [10,14,60], our aim was to determine whether FMO1 is also engaged in SAA.

Our findings suggest that the expression of *FMO1* is induced shortly after EL treatment, and that 2-minute-long exposure to EL was sufficient to induce *FMO1* expression in both local and systemic leaves. The analysis of light-induced *FMO1* expression kinetics demonstrated that its highest expression in local leaves was observed 10 minutes after the end of EL treatment. In contrast, 20 min after EL treatment the level of *FMO1* expression in local leaves dropped, but remained elevated in systemic leaves. The induction of *FMO1* expression in both local and systemic leaves was also shown for pathogen-treated plants [32,34]. These results indicate that the expression of *FMO1* is induced both locally and systemically not only during SAR, but also SAA. Interestingly, the systemic expression of the *FMO1* gene depends on phytochromes during SAR establishment, since *phyAphyB* double mutant did not increase the expression of *FMO1* in systemic leaves upon pathogen infection [61]. All these results strongly suggest the role of FMO1 in the integration of SAA and SAR signals. It has been previously postulated that in fact high-light induced SAA and SAR processes should be considered as common mechanism, for which the SAAR abbreviation has been suggested (Figure 5 and [14]).

ROS are important signaling molecules in SAA [5,8]. Therefore, in the next step we wanted to investigate if FMO1 activity has any role in the systemic propagation of ROS. Our results indicated that in wild type plants a 2-minute-long EL treatment of local leaves resulted in ROS level increase already 10 min after EL exposure. The *lsd1* mutant, known to accumulate higher level of ROS under both abiotic and biotic stress [25,26], demonstrated in our study quicker ROS propagation after local EL treatment, in relation to Col-0. This result was also confirmed by the expression level of ROS marker genes, *TRFL4*, *BGLU23* and *DLO1* that were significantly elevated in local and/or systemic leaves of *lsd1* mutant, in comparison to the wild type. In contrast, the *fmo1* mutant showed less intense ROS accumulation, compared to the wild type, which was also reflected by the expression of ROS marker genes in local and/or systemic leaves. The expression level of *DLO1*, a marker gene for H_2_O_2_ accumulation was reduced in both local and systemic leaves of *fmo1* mutant. On the contrary, the *DLO1* transcript abundance was elevated in *FMO1-OE* line. These results indicate that H_2_O_2_, which is a relatively long-lived molecule and can diffuse over long distances [62], may be the main ROS form engaged in ROS signal propagation by FMO1. However, other ROSs should not be excluded. Despite ^1^O_2_ and O_2_^−^ are rather short-lived, they can function as signal molecules, affecting many signaling pathways [8,63]. Importantly, the *lsd1/fmo1* double mutant showed similar ROS level to that of wild type, which indicates that FMO1 is a positive regulator of ROS propagation in the *lsd1* background. These results indicate that FMO1 is engaged in the LSD1-dependent ROS signaling pathway, promoting ROS accumulation (Figure 5). In this term, our results are in some way different from the study on the role of FMO1 on ROS signaling during SAR, where FMO1 seemed not to play a critical role in the regulation of the oxidative burst at the site of pathogen attack [34]. However other works examining the FMO1 involvement in biotic stresses [33,64] confirmed the role of FMO1 in ROS accumulation, observed in this study.

Taking into account that ROS are among the fastest signaling molecules, which accumulation is induced already after 2-minute-long EL treatment and the fact that *FMO1* expression can be highly induced upon O_2_^−^ generation [29], we suggest that ROS are the primary signal for activation of FMO1-dependent SAAR. However, a signaling pathway or a molecule responsible for the activation of *FMO1* expression is yet to be specified. Recent works demonstrated that the *FMO1* promoter is bound by SAR DEFICIENT 1 (SARD1) transcription factor [65], possessing redox-regulated cysteines [66]. Moreover, SUPPRESSOR OF GAMMA RADIATION 1 (SOG1) transcription factor binds *FMO1* promoter to induce its expression under oxidative stress [64]. Enhanced *FMO1* expression in response to phytotoxin inducing plant cell necrosis, thaxtomin A, in *rbohd/f* mutant, compared with wild type plants, may indicate that stress-induced extracellular ROS production is downstream of FMO1 activity [41].

Oxidative-stress induced APX2 and ZAT10 are expressed in bundle sheath cells of both local and systemic leaves in response to high or excess light and are marker genes for early SAA events [5,13,16,17]. Our results showed that the expression of *APX2* was highly elevated in EL-treated local leaves. The *APX2* induction in systemic leaves was lower than in local counterparts, but still, the *APX2* transcript abundance was increased, when compared to non-treated plants. Importantly, the levels of *APX2* expression were significantly lower in the systemic leaves of *fmo1* and *lsd1/fmo1* mutant, compared to the wild type. A similar pattern was revealed for *ZAT10*. The expression levels of *ZAT10* in systemic leaves of *fmo1* and *lsd1/fmo1* mutants were significantly reduced in relation to wild type plants. Our data are consistent with the study by Mishina and Zeier (2006), who showed that the expression levels of pathogenesis-related (PR) genes and a marker for ROS generation *GLUTATHIONE S-TRANSFERASE 1* (*GST1*) was abolished in *fmo1* systemic leaves upon pathogen attack [34]. These results indicate that functional FMO1 is needed for the induction of SAA marker gene expression in systemic leaves. Interestingly, the *FMO1-OE* line exhibited reduced *ZAT10* transcript abundance, which suggests that both lack and excess of FMO1 activity may affect transcriptional reprograming in systemic leaves.

It was demonstrated that loss of FMO1 function did not block EDS1-dependent cell death associated with the mutation in *ACCELERATED CELL DEATH 11* (*ACD11*) [67]. The *acd11/fmo1* double mutant showed similar extent of cell death to the *acd11* single mutant and it was concluded that the contribution of FMO1 to cell death initiation or execution is minimal [29]. However, the results indicating that FMO1 is a component of LSD1-dependent ROS signaling pathway prompted us to test if FMO1 is also engaged in LSD1-dependent negative regulation of cell death. In order to define FMO1 role in EL-induced cell death, we used two methods of cell death assessment, by electrolyte leakage from whole rosettes and micro-lesions counting preceded by trypan blue staining. Our results demonstrated that 3 and 6 days post-treatment the *lsd1* mutant exhibited visible symptoms of runaway cell death and significantly increased ion leakage from damaged tissue. LSD1 has been already shown to negatively regulate cell death, triggered by both biotic and abiotic stresses in the past [25,26,27]. Interestingly, our current analysis reveals that a mutation in *fmo1* reduces ion leakage in *lsd1* mutant, which indicates that FMO1 is a positive regulator of *lsd1*-dependent cell death. The expression level of *FMO1* was shown to be elevated in *lsd1* background previously and confirmed in the current study (Supplementary Figure S1), which strengthens the demonstrated contribution of FMO1 to the LSD1-dependent cell death pathway (Figure 5). LSD1 has been recently demonstrated as a transcriptional regulator [47]. Although the *FMO1* promoter was not among LSD1-bound sequences [47], it is possible that LSD1 inhibits *FMO1* expression in an indirect manner, through another transcription regulator.

Micro-lesions constitute small lesion areas within the leaf tissue, comprising one or a few dead cells [58]. Even though the development of micro-lesions in systemic leaves was previously shown during SAR in response to pathogen attack [58,59], so far there was no evidence of micro-lesion formation in the development of SAA. Here we show that micro-lesions could be observed already 24 h after 10-minute-long EL treatment and interestingly, they were generally more expressed in systemic than in local leaves. As expected, the number of micro-lesions was significantly higher in both local and systemic leaves of *lsd1* mutant, when compared to the wild type. Even though the *fmo1* mutant did not show any changes in the number of micro-lesions in local leaves, which was also shown for pathogen treatment study [34], it demonstrated significantly reduced cell death in systemic leaves. It indicates that the functional FMO1 is needed for the progression of cell death in systemic leaves. Moreover, the mutation in *fmo1* reverted the cell death in local and systemic leaves of *lsd1*, once again confirming the role of FMO1 as a positive regulator of *lsd1*-dependent runaway cell death. Interestingly, the *FMO1* transcript levels were not changed in the constitutive defense mutants, engaged in ET (CONSTITUTIVE TRIPLE RESPONSE 1, CTR1) or SA signal transduction pathway (MAP KINASE 4, MPK4), indicating that FMO1 is not activated simply by deregulated ET or SA signaling [29]. Instead, FMO1 may influence the components downstream of the LSD1-dependent signaling pathway.

In this study, we revealed that FMO1 plays an important role in SAA, which adds another evidence that EL stress-triggered SAA and pathogen-induced SAR are tightly connected and use the same signaling pathways. FMO1 functions in the induction of both SAA and SAR in treated tissue and in the propagation of a signal to distant tissues. However, its role in the perception of some long-distance signal in systemic tissue and the potentiation of defense response in systemic tissues should not be excluded. FMO1 was shown to be expressed in the tissues adjoining the vasculature [29]. Taking into account that the bundle sheath cells, surrounding vascular bundles are strongly suggested to transduce SAA signal [10], the FMO1-dependent perception and signal transmission is likely to occur in bundle sheath parenchyma cells.

## Supplementary Materials

The following are available online at https://www.mdpi.com/2073-4409/9/10/2163/s1, Figure S1: Relative *FMO1* expression level, Figure S2: Relative expression of different ROS forms markers, Figure S3: Rosette morphology of the wild type (Col-0), lsd1, fmo1, lsd1/fmo1 mutants and *FMO1-OE* line before, 3 and 6 days post EL treatment, Table S1: Primers used for FMO1 cloning and qPCR.
