# Supplementary material for: FMO1 Is Involved in Excess Light Stress-Induced Signal Transduction and Cell Death Signaling
Source: Cells. 2020 Sep 24;9(10):2163. doi: 10.3390/cells9102163 (PMC7600522; doi:10.3390/cells9102163)
Supplement: Supplementary file 1 [file cells-09-02163-s001.pdf]

Supplementary Data

FMO1 is involved in excess light stress-induced signal transduction and cell death signaling

Weronika Czarnocka [1,2*], Yosef Fichman [3], Maciej Bernacki [2,4], Elżbieta Różańska [1], Izabela Sańko-Sawczenko [1], Ron Mittler [3,5] and Stanisław Karpiński [2]

[1] Department of Botany, Institute of Biology, Warsaw University of Life Sciences, Nowoursynowska 159, 02-776 Warsaw, Poland

[2] Department of Plant Genetics, Breeding and Biotechnology, Institute of Biology, Warsaw University of Life Sciences, Nowoursynowska 159, 02-776 Warsaw, Poland

[3] The Division of Plant Sciences and Interdisciplinary Plant Group, College of Agriculture, Food and Natural Resources, Christopher S. Bond Life Sciences Center University of Missouri, Columbia, MO, USA

[4] Institute Of Technology And Life Sciences, Falenty, Al. Hrabska 3, 05-090 Raszyn, Poland

[5] Department of Surgery, University of Missouri School of Medicine, Columbia, MO, USA

* Correspondence: weronika_czarnocka@sggw.edu.pl

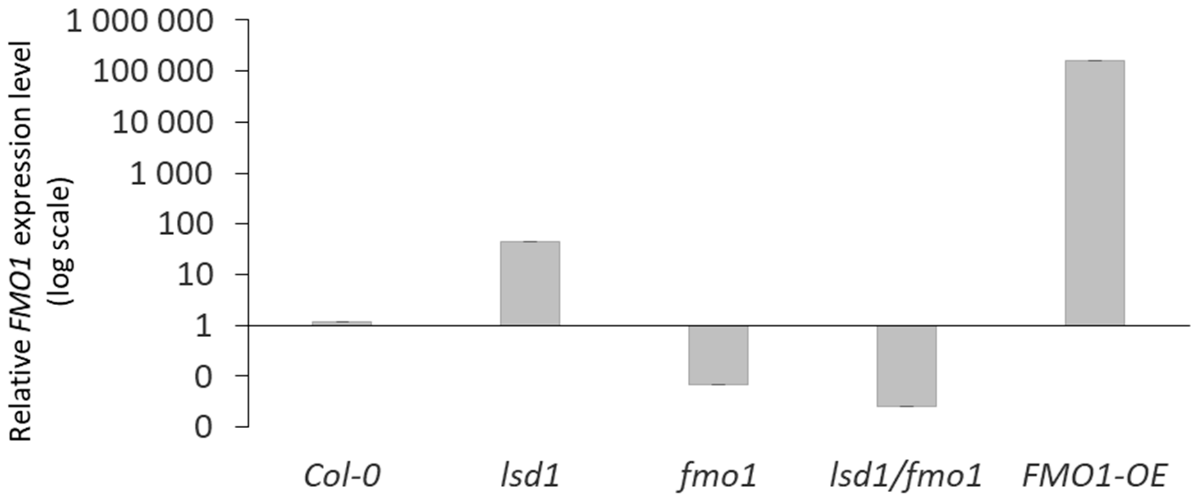

Supplementary Fig. 1. Relative *FMO1* expression level in the wild type (Col-0), *lsd1, fmo1*, *lsd1/fmo1* mutants and the line overexpressing FMO1 (FMO1-OE).

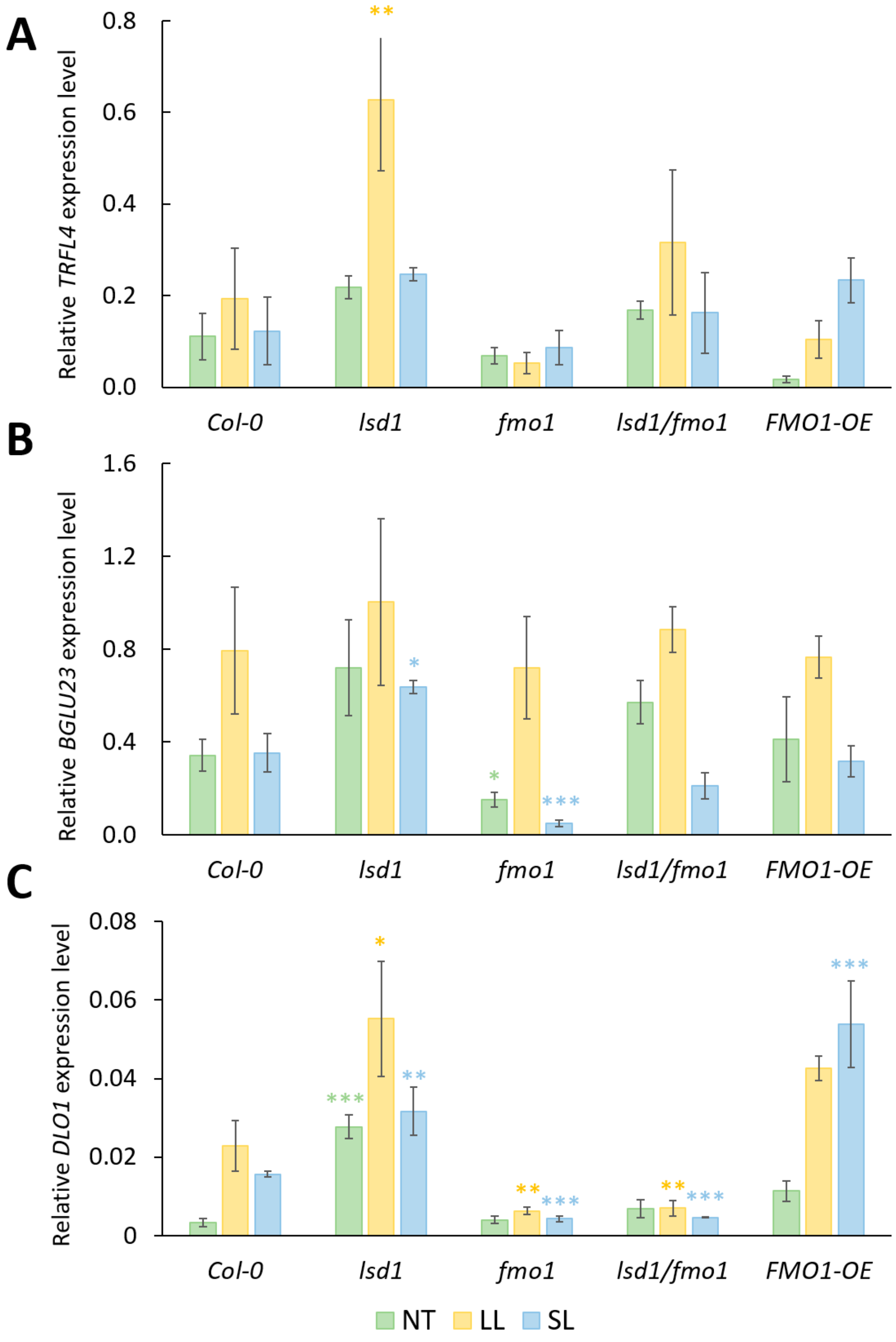

Supplementary Fig. 2. Relative expression of different ROS forms markers, *TRFL4* ($^1O_2$), *BGLU23* (O_2^-) and *DLO1* (H_2O_2) in the wild type (Col-0), *lsd1*, *fmo1*, *lsd1/fmo1* mutants and the line overexpressing FMO1 (FMO1-OE), performed for non-treated (NT) leaves, local leaves exposed to EL stress (LL) and systemic leaves (SL). Values ± SD are averages for 5 independent biological replicates, for which qPCRs were performed in duplicate (n=10). Stars above the bars indicate statistically significant difference, in comparison to the wild-type, according to the t-test at a level of $P < 0.05$ (*), $P < 0.005$ (**) or $P < 0.001$ (***).

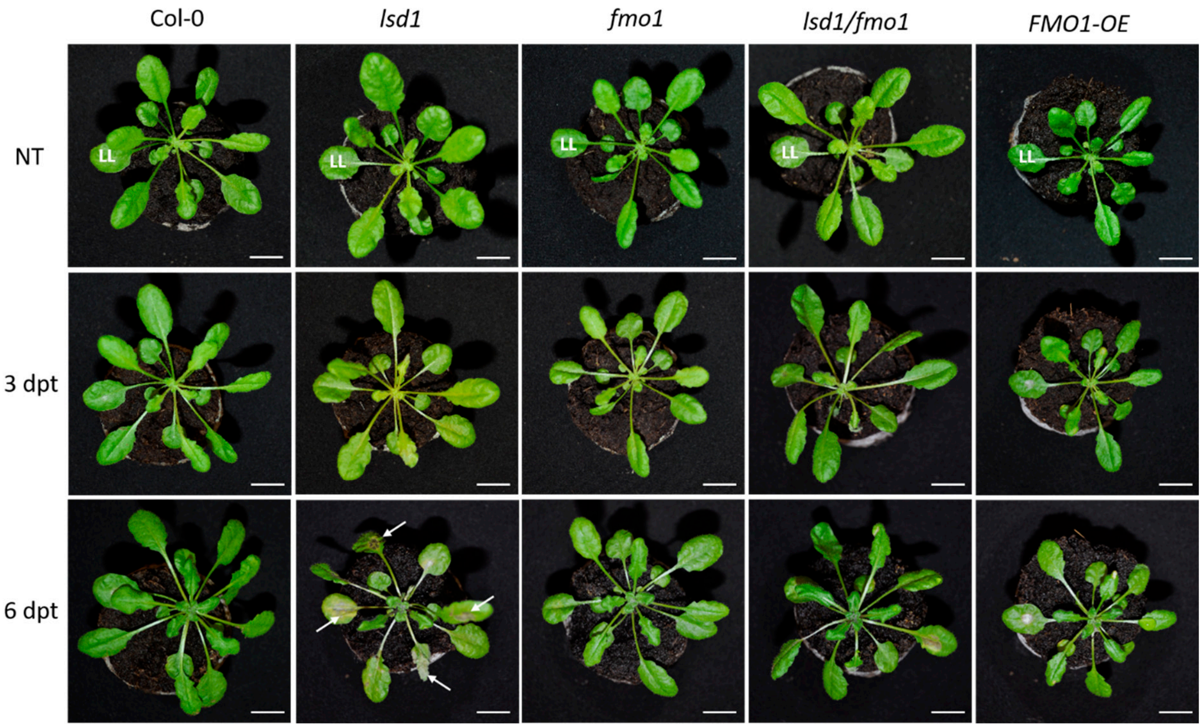

Supplementary Fig. 3. Rosette morphology of the wild type (Col-0), *lsd1*, *fmo1*, *lsd1/fmo1* mutants and FMO1-OE line before, 3 and 6 days post EL treatment.

Supplementary Table 1. Primers used for FMO1 cloning and qPCR.

Gene name	AGI code	Forward primer	Reverse primer	Application
FMO1	AT1G19250	GGGGACAAGTTTGTACAAAAAAGCAG<br>GCTCCATGGCTTCTAACTATGAT	GGGGACCACTTTGTACAAGAAAGCTGG<br>GTCTTAAGCAGTCATATCTTCTTT	cloning
PP2AA2 (Ref)	AT3G25800	TGCAATGGTTACAAGACAAGGTT	GCATTGCCCATTCAGGAC	qPCR
5-FCL (Ref)	AT5G13050	GCAAACTCAATGAACATTTTGG	GATCGGTTCATCTGCTTGC	qPCR
FMO1	AT1G19250	TGATGTTGTGATACTTGCTACTGGT	GGATGGATTGTTCCCCTGTA	qPCR
TRFL4	AT3G53790	CATTTAACCATGTAAATCATCGTACC	CTCTCCTTTGCCTTGCTGA	qPCR
BGLU23	AT3G09260	AACACAGACGCCTTTAGAATGTC	GAATTGCACACCAGCTTGAC	qPCR
DLO1	AT4G10500	CCCAACCGTTATCACTGTCC	AGCGACCCATTTATCATCCTT	qPCR
APX2	AT3G09640	TCATCCTGGTAGACTGGACAAA	CACATCTCTTAGATGATCCACACC	qPCR
ZAT10	AT1G27730	CCTACCACTTGTCACGCAAC	CTGAAGAAGAAGTAATGAAAGTGTGTG	qPCR
